# The Rohingya refugees: a conceptual framework of their psychosocial adversities, cultural idioms of distress and social suffering

**DOI:** 10.1017/gmh.2021.43

**Published:** 2021-12-17

**Authors:** Nivedita Sudheer, Debanjan Banerjee

**Affiliations:** 1 Department of Psychiatry, Christian Medical College, Vellore, India; 2 Department of Psychiatry, National Institute of Mental Health and Neurosciences (NIMHANS), Bangalore, India

**Keywords:** Asylum seekers, culture, distress, minority stress, psychosocial, refugees, Rohingya

## Abstract

Rohingya refugees, a group of religious and ethnic minorities, primarily reside in the South Asian nations. With decades of displacement, forced migration, limited freedom of movement, violence and oppression, they have been termed by the United Nations (UN) as the ‘most persecuted minority group’ in world history. Literature shows an increased prevalence of psychiatric disorders such as depression, anxiety, post-traumatic stress, insomnia, etc., in this population. However, beyond ‘medicalisation’, the psychosocial challenges of the Rohingyas need to be understood through the lens of ‘social suffering’, which results from a complex interplay of multiple social, political, environmental and geographical factors. Lack of essential living amenities, poverty, unemployment, overcrowding, compromised social identity, and persistent traumatic stressors lead to inequality, restricted healthcare access, human rights deprivation and social injustice in this group. Even though the United Nations High Commission for Refugees (UNHCR) has taken a renewed interest in Rohingya re-establishment with well-researched standards of care, there are several pragmatic challenges in their implementation and inclusion in policies. This paper reviews these multi-dimensional psychosocial challenges of the Rohingyas by synthesising various intersecting conceptual models including minority stress, health-stigma-discrimination framework, refugee ecological model and capability approach. Furthermore, it highlights multidisciplinary interventions to mitigate these adversities, improve their living situation and eventually foster healing via means which are culturally relevant and contextually appropriate. These interventions need to involve various stakeholders from a human rights and dignity based lens, including the voices of the Rohingyas and supported by more research in this area.

## Introduction: lived challenges of refugees and asylum seekers

The United Nations High Commission for Refugees (UNHCR) defines refugees as ‘people who have fled war, violence, conflict or persecution and have crossed an international border to find safety in another country’ (UNHCR, n.d.-a). Refugees are protected by international law. The Refugee Convention (1951), a key legal document, defines a refugee as ‘someone unable or unwilling to return to their country of origin owing to a well-founded fear of being persecuted for reasons of race, religion, nationality, membership of a particular social group, or political opinion.’ Global Trends in Forced Displacement-2020 by the UNHCR estimated the number of refugees worldwide at 26.4 million, among which 20.7 million are under the UNHCR mandate (“UNHCR Global Trends – Forced Displacement in, 2020,” n.d.). Based on the same report, 82.4 million individuals have been forcibly displaced globally at the end of 2020 due to conflict, persecution, violence, human rights violations and events disturbing public order (UNHCR, 2020). With war, civil unrest and political persecution enduring, thousands more join this list each year.

Often the terms ‘refugees’ and ‘migrants’ are used interchangeably; however, the UNHCR notes that these are not necessarily synonymous. The important distinction lies in the fact that the former is ‘forced’ to flee their countries of origin where their safety is threatened and seek refuge in a foreign land, while the latter ‘choose’ to move to foreign lands to improve their lives. (Refugees, n.d.-b). While there are similarities between these two sets of people, an important difference lies in the fact that immigrants have the choice of returning to their native country. When they do so, they will be reinstated with the protection of the government.

On the other hand, for refugees returning means being subject to turmoil and persecution, yet again (Refugees, n.d.-b). Conceivably, refugees are subjected to more trauma prior to migration, and subsequently as well, in the form of uncertainty over the asylum application, threat of deportation, and reduced integration with the host community (Kronick, 2018). Hence although these two populations have several similarities, their socio-ecological contexts are vastly different. Thus the ensuing challenges faced by these two populations are quite contrasting.

That migration is a risk factor for mental illness is a well-established fact in modern psychiatry (Bhugra, 2015). In a landmark paper in 2004, the author spoke of the long-term effects of migration on mental health (Bhugra, 2004). Although migrants are subject to more stress than the native population, the author argues that migration affects an individual in a nuanced manner and at multiple hierarchical levels. This results in an interaction between the individual and a complex sequence of environmental events which occur prior to, during and following migration. These events successively influence the mental health of migrants.

A review in 2015 dwelt further on these ‘pre-’ and ‘post-migration’ factors (Bogic *et al*., 2015). Among pre-migration factors, exposure to war and trauma accounted for a substantially increased risk of mental disorders. Several post-migration factors were also studied, such as unemployment, lack of proficiency in the native language of the host nation, unemployment, lack of social support and low income. There was a high prevalence of depression, post-traumatic stress disorder (PTSD), and anxiety disorders in the migrants on long term follow-up. Although studies reported varying prevalence rates, depending on where they were done, and the methodology adopted, the prevalence of these disorders typically averaged at about 20% (Bogic *et al*., 2015). One can infer from these studies and deduce that refugees are at as much, if not more risk than migrants.

## The Rohingya community: A premise

The Rohingyas are a group of religious and ethnic minorities, predominantly Muslim, native to the Rakhine state (also known as the Arakan) of a Buddhist majority Myanmar. Historical accounts show that they inhabited this region even prior to the British East India colonisation. However, the new Myanmarese government passed legislation in 1982 excluding these natives of the Arakan state from citizenship (Majeed, 2019).

Persecution from the majoritarian regimes within their country has left these people stateless (Milton *et al*., 2017) and bereft of human rights (Blackmore *et al*., 2020). Further, Myanmar's changing political scenario and the predominant military regime have allegedly persecuted these minorities under operations such as ‘Operation King Dragon’ and ‘ Operation Clean and Beautiful State’, which have led to en masse exodus since the early 1990s. With the heightening of this conflict in 2017, the United Nations (UN) agency in 2018 approximated that about 671 000 Rohingya refugees had since fled Myanmar to neighbouring Bangladesh alone. Others have sought refuge in Thailand, Malaysia, Indonesia, and India (Mahmood *et al*., 2017; Majeed, 2019; Tay *et al*., 2019*b*). These individuals have suffered decades of discrimination, violence and persecution in Myanmar (previously Burma) and are one of the largest ‘stateless’ populations globally.

A report by Mahmood and colleagues estimates that one in seven stateless refugees at present times is a Rohingya (Mahmood *et al*., 2017). Bangladesh's Cox's Bazaar district houses the largest number of Rohingya refugees (Mahmood *et al*., 2017). The camp of Kutupalong alone is estimated to house more than 600 000 people, cramped into an area of about 13 square kilometres (*UNHCR – Rohingya Emergency*, n.d.).

52% of these refugees living in Kutupalong and Nayapara refugee camps in Cox's Bazar are women and children (UNHCR-Rohingya emergency, n.d.). It comes with little surprise then that these debased families live in overcrowded shelters, often having to share it with more than one family. It is said that in these refugee camps, 93% of the population live below the UNHCR emergency standards, which are set at 45 square metres per person. Moreover, this region of Bangladesh is prone to severe monsoons with flooding, landslides and cyclones, adding the element of natural calamity to an already precipitous living environment. Thus, there is high morbidity from infectious diseases and malnutrition besides insufficient sanitary and hygiene facilities (Rohingya Refugee Emergency at a Glance, n.d.).

Over the years, much international concern has been cast on the physical and mental well-being of the Rohingyas, and effort is ongoing towards the betterment of their condition. There are existing reviews in the field looking at health and social factors (Mahmood *et al*., 2017), therapeutic strategies (Tay *et al*., 2019*a*), primary mental healthcare (Tarannum *et al*., 2019) and trauma-stress models (Riley *et al*., 2020). A systematic review by Tay *et al*. (2019*a*, 2019*b*) also summarises the cultural and psychological factors of distress (Tay *et al*., 2019*b*). Then why the need for another review?

Here the authors attempt to summarise the unique psychosocial challenges of this vulnerable population through the lens of social suffering and minority stress, outline the efforts already in place and project a roadmap for the way forward. The various biological, psychological, social, ecological and cultural factors are brought together synthesising various conceptual models of their social suffering. We hope that this not only leads to the multi-dimensional understanding of the psychiatric morbidity in the Rohingya population but also highlights them through the lens of justice and human rights. In the absence of a significant number of empirical studies in this field, the paper takes a narrative approach. No specific search strategy was employed but rather all studies pertaining to the mental health, social and cultural concerns within the Rohingya population were included for this review. Pubmed, Google Scholar, PsychINFO and Scopus were the databases searched. Certain aspects of this discussion are theoretical propositions that are extrapolated from the existing literature. There are numerous dimensions to the Rohingya crisis being referred to as the ‘most persecuted minority group’ globally by the United Nations. This review is focused mostly on the psychosocial and cultural aspects of the crisis. The socio-political, anthropological and economic connotations in the Rohingyas though briefly alluded to, are beyond the scope of the review.

## Marginalisation and human rights crisis in the Rohingyas

The state of Myanmar is yet to fully recognise the rights of Rohingyas. The Rakhine state remains one of the most underdeveloped areas of Myanmar. The government of Myanmar considers them Bengali immigrants; thus, these people find themselves ‘stateless’. Restrictions placed on them include limitations on marital and childbearing rights. Successive regimes have marginalised these minorities by impeding their access to education and employment, described in the vignettes below. Their freedom of movement is also curtailed, drawing heavy fines if they venture out of their villages without prior authorisation (Mahmood *et al*., 2017). A study by Mahmood *et al*. (2017) noted that access to aid and healthcare services provided by international agencies such as the UN and the Médecins Sans Frontières (MSF) is restricted and fluctuates with the prevalent sociopolitical climate. Rohingya children in Myanmar suffer from high malnutrition rates, which in turn makes them susceptible to infectious disease, while obstetric care to expectant mothers is limited. The authors quoted a 2014 Myanmarese government report which estimated that in Rohingya predominate townships, there was one physician per 158 000 people while in non-Rohingya predominate areas, the figure was one physician per 681 persons (Mahmood *et al*., 2017). The UN High Commissioner for Human Rights in September 2020 spoke of the continued human rights violations in Myanmar perpetrated against the Rohingya population (Schlein, 2020). She highlighted the rampant destruction of civilian property, torture and custodial deaths and extrajudicial killings of these natives of the Rakhine state. Recent satellite images also show parts of the Rakhine state having been burnt down (‘UN Warns of ‘Further War Crimes’ against Rohingya in Myanmar,’, 2020). The Myanmarese government has also subjected the Rohingya to economic-political subjugation. The government is said to have restricted access of Rohingya children to education. This has created an illiteracy rate of nearly 80% amongst them. Ever since their independence from British- colonial rule, the Rohingya were banned from military service. Those in the civil services were replaced and their political rights restricted. However, since the 1990s, Rohingya leaders have been imprisoned and their political party banned, further curbing their voices and subterfuging their chances at emancipation, within their own country (Majeed, 2019). Furthermore, global events over the past two decades have fostered discrimination along religious lines, more specifically an air of anti-Muslim racism or *Islamophobia.* The Rohingya being predominantly Muslim, Islamophobia is thought to have stoked much of the animosity against them. Bakali in his paper on Islamophobia in Myanmar, speaks of dialectical Islamophobia. He describes dialectical Islamophobia as the situation where there is an interrelationship between structural (state-mandated) and private Islamophobia. Private persons embolden by the state's stance, and amidst an aura of fear propagated by the state against Muslims, take matters into their own hands by imposing measures such as a boycott of Muslim businesses. Buddhist extremist groups are said to have added to the discrimination against the Rohingya. Moreover, the former democratic government is also accused of turning a blind eye to their fate. The author also suggests that the neglect of provision of healthcare to Rohingya predominant areas, and the restriction of their reproductive rights was perpetuated by unfounded fears of ‘Islamisation’ of what is otherwise a Buddhist majority nation (Bakali, 2021). Stories from fleeing refugees stand testament to this grim ground reality. A case report speaks of a refugee woman who failed to disclose her pregnancy to authorities as mandated. Subsequently, the woman's husband was allegedly murdered, and their property burnt down, causing her to flee Myanmar. During her subsequent time in a refugee camp, the ensuing perils lead her to lose her child only a couple of days after birth (Mahmood *et al*., 2017; Milton *et al*., 2017). Reports show that this is not a case in isolation, and that the Rohingyas' pathway to asylum is fraught with peril. Recent maritime misadventures involving the human trafficking of Rohingya refugees fleeing Myanmar has attracted much global attention. Dangers faced by fleeing Rohingyas include facing the elements, overcrowding on vessels, starvation and the risk of repatriation if caught by the authorities. In these dire situations, they also find themselves at the mercy of traffickers, often sold into slavery and flesh trade (Mahmood *et al*., 2017).

## Psychosocial challenges among the Rohingya population: peering through the medical lens and beyond

As with any other refugee population, the Rohingyas struggle with a traumatic past, diabolical present and an uncertain future. Even after fleeing persecution in their native land, they continue to endure hardships. Much of the refugees living in neighbouring countries are undocumented. A recent study by Milton and colleagues found that there were nearly 63 000–80 000 Rohingya living in Bangladesh as undocumented refugees (Milton *et al*., 2017). The authors reviewed documents on the Rohingya crisis, visited registered refugee camps, collected reports, and conducted several meetings with various stakeholders involved in the Cox's Bazar district, Bangladesh. Within these refugee camps, access to healthcare and education is limited to the registered refugees alone. Available data regarding other welfare parameters are pessimistic; the amount of potable water available per person ranged from 16 to 18 litres/day, and the number of refugees using a single latrine was around 16–20. There is a scarcity of fresh food as well. Unemployment is rampant in camps, and these camps prohibit the refugees from leaving the camp to seek work. Workers are paid less than the standards for minimum wage in these areas (Riley *et al*., 2017). More recently, a report by the Human Rights Watch, an international, non- governmental organisation, details how perilous life on the new silt island-turned refugee camp of Bhasan Char could potentially be. Located off the coast of Bangladesh, in the Bay of Bengal, this new island camp was created to overcome the overcrowding of Cox's Bazar. Authorities claim that refugees were given the ‘option’ of ‘voluntarily’ moving to this new camp, however, reports from the ground suggest otherwise. Moreover, this island is in an area that is frequently subject to cyclones and storms. There is much concern that facilities aren't adequate to evacuate the population here, in the event of an impending catastrophic storm. Furthermore, even in this new encampment, there are reports of food shortage, few employment opportunities and insufficient health care facilities. Infective disease outbreaks are commonplace and tend to overwhelm the healthcare infrastructure. Like in their native, here as well there is a restriction on the freedom of movement and those attempting to flee are taken to task by authorities. This has earned Bhasan Char the unenviable epithet of ‘*an island jail*’ (Ganguly, 2021; Human Rights Watch, 2021).

While the burden from communicable disease is the maximum, the mortality and morbidity from psychiatric illness seem comparable to that from non-communicable diseases. Among psychiatric illnesses, a majority is accounted for by psychosis and epilepsy, though systematic data on the prevalence of depression and anxiety is lacking (Milton *et al*., 2017).

A comprehensive review by Tay *et al*., in 2017 attempted to provide a systematic review of the mental health crisis faced by the Rohingya. The study estimated a high prevalence of depression (89%) and PTSD (36%) (Tay *et al*., 2019*b*). Parnini *et al*. (2013) has reported that 13% of Rohingya individuals have suicidal thoughts from a general community sample of Bangladesh (Parnini *et al*., 2013). In yet another cross-sectional sample taken from two Rohingya settlements in Bangladesh, the authors found the subjective quality of life was rated as ‘very poor’ or ‘neither poor nor good’ by an overwhelming 95% of the participants (Hossain *et al*., 2020). This is not surprising given how apart from the stressors of war and conflict and having to live in foreign lands, refugees have to also face ‘daily stressors’. These daily stressors include factors that are not directly linked to war and trauma but are prevalent in these refugee camps, including paucity of basic necessities such as food and shelter, overcrowding and unemployment. Other such stressors include concerns over one's safety and privacy. Such daily stressors have been shown to independently lead to poor mental health outcomes. Moreover, these stressful factors seemed to have a greater bearing on depressive symptomatology than a history of trauma. (Riley *et al*., 2017, 2020; Joarder *et al*., 2020).

In ‘Forced Migration of Rohingya: An Untold Experience’, the authors report qualitative research findings from 3221 Rohingya households within the makeshift camps of Cox's Bazar, Bangladesh. Perceived discrimination, witnessing neighbour's death/injury, witnessing others' homes being burnt and witnessing friends/relatives being sexually assaulted was reported by 93, 97, 85 and 59%, respectively. 115 000 Rohingya individuals were allegedly beaten, whereas 42 000 of them received gunshot wounds. An estimated 18 500 adolescents and women were raped. More than half of the Rohingya refugees ate two types of food (rice and vegetable) only inside the camp, and almost all of them suffered from malnutrition. More than 96% of the individuals demanded citizenship rights for re-establishment in Myanmar (Habib *et al*., 2018). Further, female gender, older age and repetitive daily stressors have been shown to be positively correlated with the risk of depression and PTSD (Hossain and Purohit, 2018). Gender-based violence is also quite high among them, averaging around 8–12%, despite many experts suspecting that such figures are an underestimate owing to fear, and stigma among the victims (Riley *et al*., 2017).

The same study by Riley and colleagues also found that more than 90% had witnessed violence while around 50% of the interviewed sample had been exposed to torture and violence. These repeated waves of the humanitarian crisis in the Rohingyas induced by mass displacement, violence and turmoil in daily living have been termed as ‘ethnic cleansing’, which form a major risk for PTSD and other stress-related disorders (Beyrer and Kamarulzaman, 2017).

Apart from the well expounded mental health concerns of PTSD and depression, studies also report high levels of unexplained somatic symptoms, including body pain, headache and back ache, and gastrointestinal symptoms. Also, many respondents express mental health concerns via local idioms of distress, such as being the victims of black magic or being processed by an evil spirit (Riley *et al*., 2017; Dyer and Biswas, 2019).

As a consequence of the psychological morbidity, the studies looked at the resulting functional impairment. Over half the respondents subjectively reported significant functional impairment, and health concerns were thought of as the main cause of functional impairment.

In another study by the MSF, who play a key role in delivering healthcare to this population, the MSF Mental Health and Psychosocial Team (MHPS) programme was proposed to deal with the unmet needs of this marginalised population (Dyer and Biswas, 2019). The project was to consist of two teams, each with a psychiatrist, psychologist and psychosocial counsellor, to serve two MSF out-patient clinics that were already serving nearly 170 000 individuals in seven camps. Both the refugees and primary health workers/volunteers working with them were trained in the areas of community awareness, psychiatric case identification, encourage help-seeking, reduction of stigma, basic problem-solving skills, communication, psychological first-aid and reproductive health. The authors found more than 1/3^rd^ of those attending their out-patient clinic suffered from psychosis, with a lot of these patients have had a long duration of untreated psychosis. About 26% of those surveyed during the course of this study had received prior psychiatric treatment as well. Anxiety disorders were next, accounting for around another third of the cases. A large majority of those who needed treatment for mental health concerns had received medications from pharmacies or were attended to by spiritual healers. This study also found a low follow up rate (Dyer and Biswas, 2019).

Yet another recent study done in the post-COVID-19 pandemic era, targeted at older adults amongst the Rohingya, found the prevalence of depressive symptoms to be 42%, with a higher prevalence amongst the women. This compares well with previous research done in other refugee populations such as the Bosnian and Syrian refugees, which too showed a preponderance of depressive symptoms in elder women (Mistry *et al*., 2021).

While mental health morbidity is quite high, help-seeking behaviour among the Rohingya is quite low. Research shows that less than half the population is aware of the availability of mental health resources (*Mental Health and Psychosocial Support – UNHCR|Emergency Handbook*, n.d.). Reasons for not approaching mental health services range from shame, stigma to more geopolitical issues such as language barriers, lack of documentation and inability of western relief missions to understand the local idioms of distress (Dyer and Biswas, 2019; Tay *et al*., 2019*b*).

This leads to more reliance on culturally appropriate traditional healing approaches. Religion and spirituality have been shown to be a primary source of strength and resilience for the Rohingyas (Shakespeare-Finch *et al*., 2014). Severe mental disorders, epilepsy and dementia, are considered to be ‘socially unacceptable behaviours’ and often perceived as ‘punishment for their sins’(Tay *et al*., 2019*b*). Chen (2018) mentions different types of traditional healers among the Rohingyas, namely spiritual fortune tellers, religious scholars, holy text reciters and unlicensed practitioners of Western medicine (*In Rohingya Camps, Traditional Healers Fill a Gap in Helping Refugees Overcome Trauma*, 2018). The local communities are keen to seek their help as it involves lesser stigma, and they mainly deal with sleep problems, psychosomatic complaints, possession, depression and common physical conditions. Possession by the ‘evil eye’ (Jinn or spirit) with an insidious intent is believed to lead to developmental delay, hallucinatory behaviour, somatic complaints, appetite loss and sleep disturbances. This is often considered to be a culture-bound syndrome in the Rohingyas (Boutry, 2015), with children and pregnant women being most affected.

Yet another emerging social as well as a health concern, is the involvement of the Rohingya in the drug trade. Recent reports indicate the involvement of Rohingya refugees in illegally transporting the popular methamphetamine like drug ‘Yaba’ into Bangladesh, often even as *drug mules* (Banerjee, 2019). Although it is understood that the Rohingya take up this trade out of desperation, this drug problem, if unaddressed, will only add to the mental health morbidity of these people and further complicate their road to asylum-seeking in their new countries (Das, 2017).

## Social suffering of the Rohingyas: conceptual models of distress

It is, however, reductionistic to understand the psychosocial distress of the Rohingya population just by the prevalence of psychiatric disorders. Diagnosed depression, anxiety, sleep disturbances and PTSD are but only the ‘tip of the iceberg’. Like any other vulnerable group, the emotional and social challenges of the Rohingya individuals can be conceptualised through **‘minority stress’** that cause various dimensions of their social suffering (Prasse-Freeman, 2017). Members of stigmatised minority groups (age, gender, racial, ethnic, etc.) are exposed to long-term high levels of stress due to poor social support, low socioeconomic status, income inequality, discrimination and interpersonal prejudice. This leads to physical and mental health disparities in these vulnerable groups (Dohrenwend, 2000; Meyer, 2003). Rohingyas face a similar situation, compounded by compromised healthcare access, political violence and societal apathy (MacLean, 2019). Their socio-cultural peculiarities have specific ‘idioms of distress’ which vary in expression and are frequently unrecognised by mental health professionals (MHP) from outside their communities (Elshazly *et al*., 2019). According to Tay *et al*. (2019*a*, 2019*b*), there exists limited familiarity with ideas of mental health and counselling among the Rohingya groups, both due to a lack of appropriate information related to healthcare and feelings of shame (Tay *et al*., 2019*b*). Individuals with severe mental illness in their community are mostly taken care of by their families, and professional help is not sought due to the ‘fear of ostracization’ (Simpson and Farrelly, 2020). There also exist separate linguistic terms for various aspects of existence (brain, mind, soul, body) as well as emotional distress, suicide and depression, which are rarely appreciated outside the community. Some of these culturally specific terminologies used by the Rohingyas to describe various aspects of mind and body are summarised in Table 1. Importantly, the traditional body-mind dichotomy of the dominant psychiatric and psychological models are less accepted among the Rohingyas, and there is scant literature on this ethno-psychological idea related to mental health among them (Islam and Nuzhath, 2018). Like racial and sexual minorities, Rohingyas also suffer from marginalisation as a result of minority stress, making them susceptible to both proximal and distal stressors and, in turn to adverse health outcomes (Hossain and Purohit, 2018; Tay *et al*., 2019*a*, 2019*b*). The various facets of their psychosocial challenges are highlighted in Table 2.

**Table 1.** Culture-specific terminologies among the Rohingyas related to body and health

Category	Specific aspects of health/body	Terms used
General	Self	*ma-muish*
	Organs/body parts that can be seen	*zaa-héri-hissá*
	Organs/body parts that cannot be seen (abstract)	*baa-temi-hissá*
Body	Brain	*demag*
	Physique	*jism*
	Mind/heart	*dil*
	Soul	*foraan/poraan*
Brain functions	Thought	*tára*
	Memory	*hafé-za*
	Reason	*oujá*
	Learning	*che-khoum*
	Comprehension	*bazon*
Other cognitive abilities	Attention	*dhivan/dyan*
	Motivation	*tuaijjn*
	Sensation	*ehsaas*
Specific body-parts	Head	*matá*
	Chest	*seena*
	Limbs	*háat-gent*
	Stomach	*feeht/peet*
	Lower back	*feeson*
	Buttocks	*faasu/paasu*
	Knees	*haanthu*
Emotions	Happiness	*khúsh*
	Sadness	*pere-chani*
	Anger	*guissa*
	Love/intimacy	*adorro*
	Surprise	*taijuzbbi*
	Disgust	*chokko-gossonn*

**Table 2.** Different forms of psychosocial challenges and related stressors within the Rohingya population

Psychosocial challenges	Specific stressors
Displacement	• Compromised physical and emotional security • Social injustice • Human rights violation • Disruption of social identity • Impaired social cohesion
Forced migration	• Unemployment • Poverty • Food and housing insecurity • Reduced access to healthcare • Restricted education
Marginalisation	• Minority stress • ‘Stateless' lives • Lack of administrative onus • Age and gender-based discrimination within the community • High levels of illiteracy • Lack of health policies/social welfare • Lack of citizenship rights
Violence	• Social isolation and mistrust • Physical insecurity • Reinforces displacement and internal violence • Increased crime rates • Psychiatric comorbidities (especially depression, adjustment disorders and PTSD)
Traumatic events	• Direct: violence, persecution, imprisonment, torture • Indirect: exposure to loss, deaths, injuries and destruction • Refugee crisis
Idioms of distress	• Unique linguistic and socio-cultural norms related to mental illness • Limited understanding of the traditional mind-body model • Reduced help-seeking (stigma, shame) • Limited awareness about mental health • Restricted social integration (through interaction, education and media)

### Health, stigma and discrimination (HSD) framework

Social stigma is a well-researched barrier to treatment adherence, engagement in care and help-seeking behaviour among the Rohingyas (Reda, 2020). Based on the HSD Framework proposed by (Stangl *et al*., 2019), fear of social oppression, discrimination, authoritarianism, stereotypes and economic inequality can serve as ‘stigma drivers’ and the lack of dedicated legal/health policies, and unique socio-cultural norms can serve as ‘facilitators’ at an individual, interpersonal, organisational and community level for the Rohingyas (Ripoll, 2017; Sohel, 2017; Karo *et al*., 2018; Stangl *et al*., 2019). This can further lead to perceived rejection, internalisation of social blame, self-stigma, secondary stigma and discriminatory attitudes within the group itself (Ahsan Ullah, 2016; MacLean, 2019). These factors have been postulated as reasons for the increased within-group rivalry and violence among the Rohingyas. In the absence of social welfare and protection schemes and lack of advocacy, these factors further perpetuate social injustice, human rights violation and compromised resilience within this population. In the adverse circumstances of refuge-seeking, frequently adapting social identities and poverty, all the above-mentioned adversities can lead to survival threats which form the bulk of psychosocial challenges of the Rohingyas, besides the increased prevalence of psychiatric disorders (Elshazly *et al*., 2019; Guglielmi *et al*., 2020). There is evidence to suggest that chronic stress, deprivation, adverse early experiences and inequalities can lead to altered stress response and epigenetic changes of the hypothalamic-pituitary-adrenal (HPA) axis. Berger and Sarnyai summarise how racial discrimination leads to several neurobiological changes such as chronically elevated cortisol levels, and dysregulation of the HPA axis, thus leading to increased allosteric load and chronic disease (Berger and Sarnyai, 2015). Such persistent stress has been shown to induce epigenetic changes in refugees and persecuted individuals leading to transgenerational stress. Epigenetic changes most commonly involve the genes concerning the HPA axis, which can persist in generations living through the same socio-environmental stressors, impacting their capacities to adapt to the stress (Gudsnuk and Champagne, 2012). Studies also link social isolation with epigenetic changes, key amongst them is those associated with the BDNF gene, a key mediator of many vital bodily functions, including stress response and neuronal proliferation and plasticity (Di Carlo *et al*., 2019). Social adversity such as low socio economic status is also associated with premature onset of chronic disease and curtailed life span (Fiorito *et al*., 2017). Transgenerational stress in Asian American refugees has been shown to affect reproductive success, migration, social behaviour, survival benefits, stress response and cognitive abilities (Kwan, 2019). Hence psychobiological effects of existential stress is significant in refugees and their living.

### Ecological model of refugee distress

**Fig. 1.**
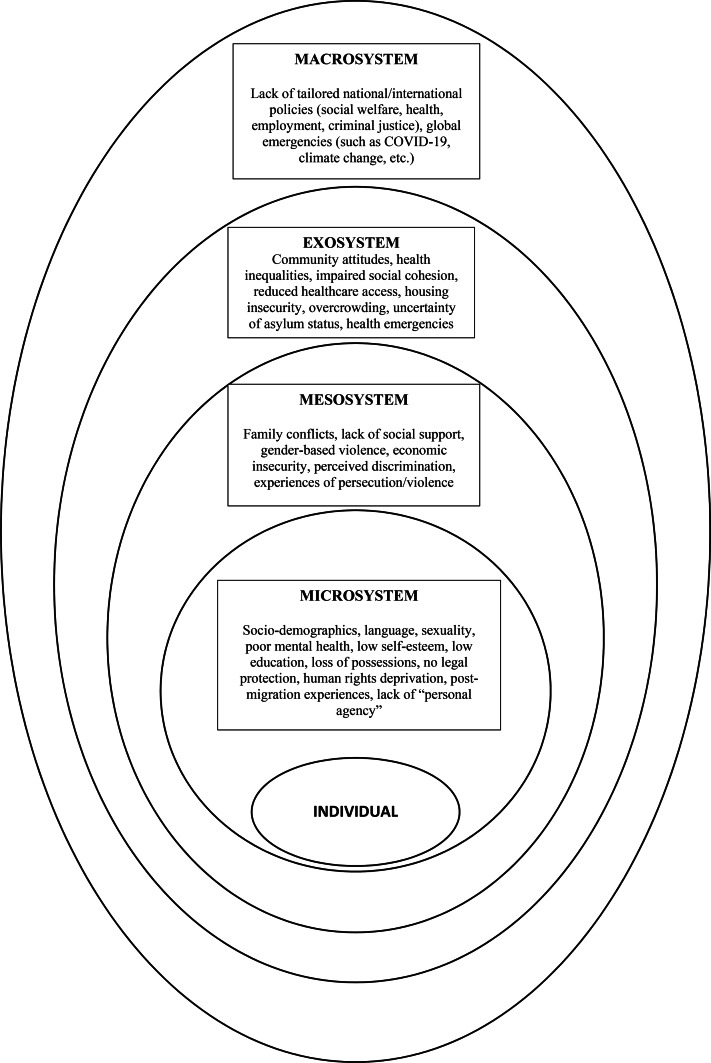
Various post-displacement stressors in the Rohingya community influencing their psychosocial wellbeing. Starting from an individual level, these stressors are depicted in different levels of their ecosystem.

The Rohingya psychosocial distress can be conceptualised through other models as well. Miller and Rasmussen (2017) had proposed this model, which looks at the persistent and strong effects of ongoing stressors related to the perceptions of displacement itself (Miller and Rasmussen, 2017). This model posits that the mental health offshoots of refugees and asylum seekers not only stem from experiences of prior conflict exposure but also from instability in their social and environmental ecology. Based on this model, ongoing daily stressors assume more importance in the Rohingyas. The crucial post-displacement related stressors in the Rohingyas at various levels are highlighted in Fig. 1. While the intensities of the experiences may vary, Miller and Rasmussen (Miller and Rasmussen, 2010*a*) had proposed four ways in which they may affect the mental health of refugees – first being temporal proximity of the displacement-related stressors, second the perceived lack of control, third the pervasiveness of these traumatic experiences and finally their diversity and uncertainty. Humanitarian aid, legal provisions, livelihood programmes, vocational skills training and work opportunities need to be aligned with psychosocial interventions keeping in mind these socio-ecological stressors (Miller and Rasmussen, 2010*b*).

### Capability approach (CA)

Lately, this model was expanded upon by White and Van der Boor (2021), who proposed ‘a human development approach to conflict and displacement-related stressors’ (White and Van der Boor, 2021). The need to explore broader psychosocial outcomes beyond the prevalence of psychiatric disorders led the authors to formulate this framework that is based on an individual's freedom, sense of agency and being able to do what is/are valuable to them. Being restricted of their autonomy and freedom form the basic modes of human rights deprivation in refugees such as the Rohingya individuals. This can be noticed at various levels: the microsystem (individual-related factors such as age, sex, impairment, etc.), mesosystem (social experiences such as responsibilities, abuse, support, etc.), exosystem (experiences in the person's network such as media portrayals, perceived discrimination, etc.) and the macrosystem (institutional level factors such as legislation and policies). The CA framework has also been used to propose multi-sectoral coordinated psychosocial interventions for the refugee populations based on dignity, autonomy and respect (mentioned later) (White and Van der Boor, 2021).

### A biopsychosocial understanding

The models discussed above are not mutually exclusive but rather intersect at various levels. Let us attempt an integrated understanding of their social suffering through the models described above. Decades of oppression, refugee status (lack of citizenship), violence, social isolation and political persecution have led to socio-economic inequalities which have been further widened by health crises such as the coronavirus disease-2019 (COVID-19) pandemic. Social stigma and rejection have also compromised healthcare access perpetuating these health inequalities (HSD model). These setbacks are further compounded by post-displacement stressors including food and housing insecurity, internal crimes, poverty, unemployment, etc, which in addition to the lack of social welfare benefits and invisibility in research maintain ‘minority stress’ within the Rohingya population. They face unique challenges in all levels of the ecosystem (Ecological model), as well as all of these challenges, lead to restriction in their movement, compromised freedom and personal autonomy that lead to helplessness and reduced ‘sense of agency’ (Capability Approach). Each of these factors is not independent in itself but intersect at multiple and complex ways to result in the increased prevalence of psychiatric and substance use disorders in the Rohingyas. This psychiatric morbidity is associated with social injustice, existential threats, mental health inequalities and restricted access to mental healthcare, all of which eventually lead to human rights crisis.

Hence, biological vulnerabilities and epigenetic changes are but expected within a community with decades of ‘minority stress’. The models of social suffering add to the emotional and social components, thus extending the classical biopsychosocial understanding of mental disorders in them to involve cultural and ecological components as well.

**Fig. 2.**
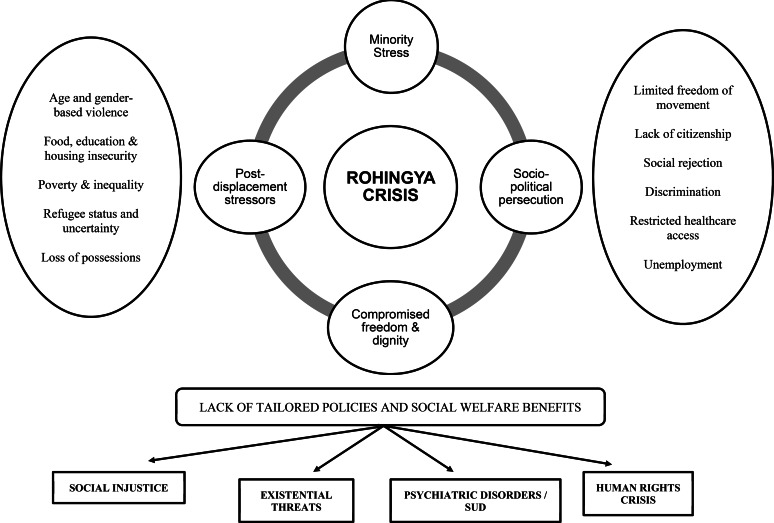
Conceptualisation of the psychosocial crisis of the Rohingyas through a synthesis of various interlinked models (minority stress hypothesis, health-stigma-discrimination framework, post-displacement and ecological stressors, capability approach). Various dimensions of stress contributing to all these models are depicted on both sides. The cumulative effect of all these stressors leads to a biopsychosocial vulnerability for the Rohingyas (increase in psychiatric disorders, substance abuse, survival threats and human rights crisis). (Miller and Rasmussen, 2017; Prasse-Freeman, 2017; Stangl *et al*., 2019; White and Van der Boor, 2021). SUD: Substance Abuse Disorders.

In an attempt to depict these intersections, Fig. 2 provides an overview of the psychosocial distress within the Rohingya community that includes principles of minority stress, health stigma and discrimination, post-displacement stressors and capability approach.

## A double hit: effects of the COVID-19 pandemic

Bangladesh is one of the most population-dense countries in the world, and the COVID-19 pandemic has struck the nation, viz a viz the one million refugees hard. Overcrowding and lack of potable drinking water have made these refugee camps ideal for propagation of the causative severe acute respiratory syndrome coronavirus 2 (SARS-CoV-2). Lack of adequate personal protective equipment (PPE) and face masks have compounded the problem of delivering aid in these areas (Banik *et al*., 2020). Despite this major threat, the WHO, along with the Bangladeshi government, have worked together to mitigate damage, and in about 1 year since the outbreak of the pandemic, had reported only ten deaths across all refugee camps (Khetrapal Singh, 2021). As with the rest of the world, the pandemic has amplified other deficits such as food shortages, access to education, deficiencies in obstetric care and non-communicable diseases. Lockdown and the ensuing shutting down of local businesses have left many(around 85%) unemployed. Here as well instances of gender-based violence were on the rise (Balakrishnan, 2021). In terms of psychological morbidity, reports suggest that an increase in depression, anxiety and sleep disturbances in the uncertain times created by the pandemic were seen (Masudur Rahman *et al*., 2020). In Mistry *et al*.,s' study (2021)on older adult Rohingyas, they found that a struggle to access food, routine medical care and medication during the pandemic were likely to lead to depressive symptoms. Additionally, the fear of contracting COVID and associated morbidity were important causes of psychiatric morbidity (Mistry *et al*., 2021). Building on the social stress theory, the pandemic has exacerbated the stressors endured by this population (Campo-Arias and De Mendieta, 2021). A comprehensive public health programme has been called upon for special refugee populations, such as the Rohingyas, with a need to address their enhanced vulnerabilities during the COVID-19 crisis. Medical protection measures, awareness about the illness, facilitation of social distancing and availability of basic amenities needed for survival are the suggested measures (Alemi *et al*., 2020). And now in a post- COVID 19 vaccination era, the stark rift between the Rohingyas and the rest of the world is again highlighted. While the rest of the world is attempting to surge ahead, the Rohingya wait down a long list for their turn to get vaccinated (Ahasan, 2021).

## Revisiting the existing service provisions

Despite many challenges, since 2017, government and non-government agencies have tediously worked for the betterment of these stateless persons (Tay *et al*., 2019*b*). MSF data suggests that since August 2017, they have provided more than 17 000 individual consultations and in excess of 18 000 group sessions for mental health concerns. In addition to out-patient consultations, they were also able to offer in-patient facilities in a few of the health centres. Stigma and lack of awareness remain significant impediments for the refugee population to seek out mental health care. As Prodjut Roy, an MSF Mental Health Supervisor in Bangladesh noted, the Rohingya associated the term ‘mental’ with insanity and hence felt stigmatised. Instead, referring a patient who needed psychological help to the subsequently renamed ‘Shanti Khana’ (peace centre) overcame this particular impediment (*Shanti Khana*, n.d.). In addition to the MSF, the UNHCR is the other major player involved in providing psychosocial remediation for the refugees. The UNHCR has adopted ‘Mental Health and Psychosocial Services’ (MHPSS) as part of its public health policy in conflict-ridden areas. The focus of their work has been on the provision of psychiatric/psychological help, ensuring availability of essential psychiatric drugs (the list now has 12 drugs including antipsychotics, anxiolytic and anti-epileptics), education and support groups for parents of children with nutritional deficiencies, and provision of psychological intervention including Integrate Adapt Therapy and Interpersonal Therapy (Salim, 2021). Integrate Adapt Therapy (IAT) is based on the principles of the adaptation and development after trauma (ADAPT) model (Silove, 2013). The core principles of the ADAPT model are safety/security, networks, justice, roles/identities and existential meaning, each of which can be disrupted by mass conflict leading to societal instability. Compared to the existing cognitive behavioural therapy (CBT) and interpersonal interventions, IAT deals with the process of trauma and helps the individuals navigate their emotional and behavioural problems with relation to the environmental psychosocial disruptions. This novel therapy aims to provide meaning to their life experiences, and early trials have shown promising results (Tay *et al*., 2019*a*, 2020).

Tay *et al*. (2019*a*, 2019*b*), in their focus group interviews with 12 lay IAT counsellors, assessed the benefits, cultural acceptability and implementation challenges of the IAT programme. All the counsellors completed a training workshop for the same, followed by six-month supervised use for Rohingya refugees. Both the refugees and counsellors agreed on its efficacy and feasibility. Gains were noted in interpersonal relationships, community ties, purpose and meaning of lives as refugees and realistic view of their identities. Gender balance and training infrastructure were notable challenges (Tay *et al*., 2019*a*). Mahmuda *et al*. (2019) recently used Group Integrative Adapt Therapy (IAT-G) as a strategy to address the gap in MHPSS services for Rohingya individuals in Bangladesh. It has been mentioned as a ‘pragmatic, eclectic and transdiagnostic’ approach based on the ADAPT model, which targeted the maladaptive behaviour towards post-migration living and trauma as well as helped foster resilience. Psychologists and para-professionals could implement this approach in the Rohingya community (both onsite and online sessions), focusing on cultural adaptation of the sessions, digital literacy and periodic assessments. Though it was a feasible intervention, introduction and sustenance were noted as major barriers in service delivery (Mahmuda *et al*., 2019; Tay *et al*., 2021).

Another effective method has been the use of community volunteers to act as liaisons between the Rohingya people and the aid workers (Uddin and Sumi, 2019). These community volunteers have helped identify, refer and encourage the Rohingya community to enlist mental health care.

The role of media is vital in this regard as the portrayal of their crisis facilitates discourse and concerns in the national and international forums. Refugee representations in the US media show that they are often framed as ‘threatening’ and ‘dangerous’, which descriptions such as ‘fugitives’ and ‘invaders’ (Mountz and Mahtani, 2002; Marinescu and Balica, 2021). The representations also vary between the forms of resettlement – dispersal into the communities *v.* people being accommodated in camps. This leads to altered social perception, which views refugees with panic and anxiety, associating them with ‘large, helpless and desperate violent masses’ (Kaleda, 2014). It further reinforces social stereotypes where the community feels insecure due to the refugees and, in turn, deals with hostility, apathy and neglect (Baker and McEnery, 2005). One of the critical problems in refugee crisis from any community, as termed by Bleiker *et al*. (2013), is the media tendency to ‘massify’ refugee representations leading to the loss of their individual identities as persons with sufferings. They are no more identifiable victims with faces or persona but ‘just anonymous masses…an abstract and dehumanised political problem’ that absolves the society of any responsibility (Bleiker *et al*., 2013). This is detrimental to refugee threats and survival conditions. In a recent study that examined coverage of predominant news frames and refugee characteristics across newspapers from four nations, Irom *et al*. (2021) reported victim, thematic and administrative frames to be commonly used. Politicians, NGOs and internal organisations were the main sources, and though the reporting of the refugee crisis was essentially ‘positive’, personalised frames were lacking, which could have provided a ‘nuanced portrayal’ of their challenges. The authors highlight the need for journalists to consciously revisit the humanitarian angles of the Rohingya crisis and provide a socio-politically unbiased angle of their suffering (Irom *et al*., 2021).

The attempts at psychosocial care are still in the nascent stages. Coming on the heels of more existential challenges, such as fulfilling the food, shelter and security needs, the road is long and drawn to the day when adequate mental health services can be provided.

## The Indian scenario

Despite being home to refugee seekers since independence, India is not a signatory to the 1951 Refugee Convention. Hence there are no existing laws governing refugees, and laws are made ad-hoc (Vijayaraghavan, 2020). Yet, it is among the many neighbouring countries where the Rohingya have sought refuge. A search of academic literature reveals a paucity of research from India on the status and plight of this population. Considered as ‘illegal immigrants’ in India, these people are bereft of basic rights. In the absence of systematic literature, most of the data is obtained from online forums and media reports, which run the risk of bias. A 2018 newspaper report quotes the Indian Ministry of External Affairs data, which seems to indicate roughly 14 000 Rohingyas in India; however, other estimates suggest that the number (in 2018) was likely around 40 000, with these immigrants scattered in various parts of the country (Tripathi, 2018). More recent estimates place the official number at 20 000. Myanmarese refugees in India are not under government jurisdiction but fall under the ambit of the UNHCR. Hence despite their refugee status being recognised by the UNHCR, their legal status is not endorsed by the government. This translates into trouble on the ground, wherein their access to amenities falls to the mercy of local authorities or other service providers. This report by Vijayaraghavan details how this means, for instance, that certain schools may deny entry to immigrant children. Thus a lack of access to government-issued documentation can result in marginalisation (Shanker and Raghavan, 2020; Vijayaraghavan, 2020). The COVID-19 pandemic has further cast them into peril. It started with rumours in the media alleging the Rohingya as one of the initial super spreaders, which triggered stigma, xenophobia and isolation in this already vulnerable population. They also failed to get the food, financial aid extended to other migrant communities during the lockdown. Hunger, fear, lack of awareness and inaccessibility to face masks and soaps are some of the major issues plaguing these camps. Despite efforts from locals and humanitarian organisations to help and rehabilitate the refugee, much has been left wanting (Bose, 2020). As with internal migrants, the refugees too faced a loss of livelihood from the lockdown. The absence of financial or targeted medical aid to these populations left them bereft of essential supplies (Shanker and Raghavan, 2020).

This comes on the background of emerging hostility against the Rohingya (Kumar, n.d.) and reluctance on the part of the Indian government to fully commit to the Rohingyas (CNN, n.d.). Indian government officials have been frequently quoted as saying that the Rohingyas will be deported back to Myanmar (Human Rights Watch, 2017). Within India, these refugees are seen as vulnerable groups, at risk to be lured by other extremist organisations and hence under scrutiny from intelligence agencies in the country (*Rohingyas: 36 000 Rohingyas in India; Terror Links Cannot Be Ruled out: B.S.F. – The Economic Times*, n.d.; *India's Rohingya Terror Problem*, 2017). Most recently, the Supreme Court of India upheld the Government of India's decision to deport ‘illegal Rohingya immigrants’, a move that has left this community in shock and fear(Gupta, 2017; *‘Supreme Court Has Signed Our Death Warrant’: Rohingya in India|Rohingya News|Al Jazeera*, n.d.). Thus within India, the Rohingya dreams for a better future seem a long way to go and needs concerted efforts from all stakeholders.

## Gaps in research

Much of the work on studying the mental health status of the Rohingya population has focussed on trauma and PTSD, or other severe mental disorders. As mentioned before, numbers and related statistics of psychiatric disorders are only a minor part of the multi-faceted psychosocial challenges faced by them. As Riley and colleagues have pointed out, it is the daily stressors that account for much of the psychological morbidity (Riley *et al*., 2017). Not being sure of having three meals a day or concerns over one's own or loved one's safety and security, scarcity of shelter are more proximal and direct predictors of stress, which lead to an existential crisis. This again brings to the well-expounded concept of ‘minority stress’ (discussed earlier), which notes that minority populations in every society are subjected to prejudice and discrimination, which are causes of chronic stress. Exposure to minority stress is also known to be one of the contributors to epigenetic changes over time (Ng *et al*., 2019). Epigenetic changes are thought to contribute to the aetiological processes of cancer, cardiovascular disease and mental illness, and one can also only wonder about the epigenetic changes that accrue over the generations in these refugees exposed to such hardships (Vick and Burris, 2017). The future of an entire generation lies in limbo in these camps, with no identity or a certain tomorrow. With their mere existence, their identity being questioned by the powers that be, it is but certain that these individuals are far from mental peace. Nevertheless, mental health researchers have oftentimes looked at it from a ‘medicalised’ lens, which may fail to do justice to the social inequality and social suffering that they face. That health, especially mental health, is intertwined with ones' social identity cannot be ignored. The existing studies highlight a large number of sub-syndromal anxiety, depression and high levels of somatic complaints which serve as ubiquitous ‘idioms of distress’. Discounting these idioms of distress and merely focussing on established psychiatric syndromes, would be a gross-under estimation of the magnitude of the problem at hand. Existing literature illustrates quantitative data, but qualitative data is scarce. Only a few papers detail the lived experiences in a refugee camp or what statelessness truly means for an individual. Further, adverse childhood experiences (ACEs) serves as predictors of future mental health and mediators of epigenetic change is well established (Kessler *et al*., 1997). Prospective studies examining the impact of ACEs by way of life in a refugee camp, and its effect on the longitudinal health of an individual is scarce. What needs to be further understood in the Rohingya population are the biopsychosocial mechanisms in how these adversities increase psychiatric problems, the processes of coping and resilience, identify the internal strengths of the community, expressions of the idioms of distress related to mental health and help-seeking behaviour. The salient areas where further research needs to be focused on are summarised in Table 3.

**Table 3.** Possible areas of future research to address the current gaps

• Population-based studies on the prevalence of CMD and SMD • Moving beyond the categorical approach to conceptualise psychosocial distress • Explore cultural idioms of distress and social suffering • Lived experiences and perceptions of different sectors of the population • Focus on vulnerable populations within the Rohingyas (children, adolescents, older people, women, gender minorities, etc.) • Study of rehabilitation experiences • Evaluation of efficacy, implementation and challenges associated with community interventions • Effect of international politics, xenophobia and hate-crimes on their psychosocial crisis • Models of psychological resilience in different age groups/sectors • Specific challenges during the COVID-19 crisis

## Psychosocial interventions: role of the MHP

As discussed, there is much scope in the efforts to emancipate the Rohingya refugees. In many nations, the Rohingyas represent not only an under-represented population but also an untapped economic force. Improving functional outcomes in them will serve the host nation well on a socioeconomic front. This, however, is not synonymous with a neo-liberal agenda of work exploitation through this marginalised population but rather implies that social participation of the Rohingyas needs to be encouraged for their economic development and independent as well as inclusive social identities (Rosyidin, 2017). Such an approach calls for a paradigm shift from the current standpoint to collectivism. For this population to be economically viable, their day to day concerns must be addressed vis a vis, those described above as ‘daily stressors’. The role of MHP is crucial in identifying, addressing and reviewing the psychosocial concerns of this population. This cannot happen in a vacuum, and psychosocial care needs to be incorporated as an integral part of public health.

A study by the International Organisation for Migration (IOM), a UN agency in the Rohingya camps of Bangladesh, looked at the ‘mental health and psychosocial needs of the refugees’. Their assessment showed food scarcity, lack of adequate living spaces and lack of identity as among the main reasons for distress. The remedial measures recommended by this team saw a pyramid approach, with the main focus being on the provision of food, water and security. This is also recommended by the IASC guidelines, MHPSS, which serve as essential parameters for MHPs to improve community-based psychosocial health of the Rohingyas (Inter-Agency Standing Committee (IASC), 2007; *Mental Health and Psychosocial Support – UNHCR|Emergency Handbook*, n.d.). Besides health, intersectoral coordination needs to exist with those of housing, food security, sanitation, social welfare, protection, education and rehabilitation. The basic tier of services includes delivery of daily amenities including food, water and security along with gender mainstreaming and human rights preservation. Subsequent measures suggested included strengthening of the resilience in the community, group interventions for certain vulnerable sub-groups and finally liaison with local tertiary care centres for provision of specialised and equitable care (Elshazly *et al*., 2019).

Thus the approach must be both multi-modal and multi-tiered, with the Union and State governments leading the way, followed by international agencies including the United Nations, International Red Cross, the MSF and the grassroots organisations and NGOs. MHPS can form the pillars of the system, actively liaising with other services at all levels of healthcare and administration through advocacy and mental health promotion for this population. Especially in nations with limited resources where the Rohingyas reside, integrating mental health into primary healthcare is of utmost importance (Tarannum *et al*., 2019). Health-system readiness, capacity building, supervision and active communication with health policymakers are necessary for this integration and scaling up. The UNHCR used the Mental Health Gap Action Programme Humanitarian Intervention Guide (mhGAP-HIG) (WHO, 2015) to design a programme for such integrative services in Cox's Bazar, Bangladesh (Tarannum *et al*., 2019). The mhGAP-HIG is unique in the sense it provides general evidence-based principles for caring for people with mental, neurological and substance use conditions in humanitarian emergencies (WHO, 2015). These include timely communication, assessment, appropriate management, strengthening social support, stress reduction, protection of human rights and holistic preservation of well-being while dealing with any psychological disorder (contextualising the care in living conditions and social settings).In this study, sixty-two primary healthcare workers were trained (basic psychoeducation, mental state examinations, psychosocial assessment and support), who provided 1200 mental health consultations in a year's time under the supervision of a psychiatrist. A referral system has also started, from primary care to district hospitals and essential psychotropics have been made available at health centres (Tarannum *et al*., 2019). Resource utilisation and effective primary-tertiary collaboration were reported as the main pillars. The MHPs are also uniquely equipped for relevant research in the field, especially lived experiences, perceptions and resilience of the Rohingyas. This may help in tailoring policies and designing psychotherapeutic interventions to mitigate stigma, trauma and foster resilience. The various modules of this multi-tiered system are summarised in Table 4.

**Table 4.** Multi-tiered MHPSS psychosocial interventions for the Rohingya population

Tier no.^a^	Service/Intervention	Principles
1	Essential services and security	• Basic needs (food, water, housing, sanitation, healthcare, infection control) • Physical security • Preserving gender-rights • Target more vulnerable groups • Violence rehabilitation
2	Community and family support	• Restoring social cohesion among the refugees • Re-establishment • Community representation and participation based on age, gender, diversity • Building networks (ex: child-friendly spaces, refugee volunteer groups, etc.) • Family support to prevent gender violence
3	Targeted psychosocial support	• Problem-solving and practical approaches • Resilience building and coping • Psychological first-aid • Restoring identity (individual and community) • Using non-specialised health interventions (involve primary and secondary healthcare)
4	Clinical	• Equitable access • Identify and treat severe mental disorders, common mental disorders, substance abuse, psychiatric emergencies • Suicide and self-harm prevention • Mental health counselling • Psychosocial rehabilitation • Primary-Tertiary mental healthcare collaboration

MHPSS, Mental Health and Psychological Services (UNHCR, 2018).

a The tier no. in ascending order signify bottom-up orientation of services with 1 being the lowest and 4 being the highest in the order.

As mentioned before, interventions for the Rohingyas need to be focused on through the lens of social justice and human rights. There has been a constructive change in the human-rights perspective as well from ‘negative freedoms’ (ex: prevention of harm and conflict) to more ‘positive freedoms’ (agency to do and be what an individual values) (Burchardt and Vizard, 2007; Vizard, 2007). Based on the CA approach (White and Van der Boor, 2021) discussed earlier, there is a ‘reciprocal relationship’ between psychosocial functioning and human rights in the Rohingya refugees. The lack of appropriate rights-promoting legislations (macrosystem), lack of security in communities and prejudice (exosystem), abuse and neglect (mesosystem) can finally lead to impaired psychological well-being in an individual who has been forcibly displaced (microsystem). Using a similar approach, a recent study used interpretative phenomenological analysis to analyse 16 focus group discussions conducted with female refugees in the United Kingdom (Van der Boor *et al*., 2020). It was essentially an open-ended exploration of ‘what good life meant to them’. The predominant themes that emerged were personal agency, legal security and social cohesion (or connectedness). In another similar qualitative study by Chiumento *et al*. (2020) among Congolese refugees from two refugee settings in Uganda and Rwanda, the need to have a ‘self-valued and free life’, conflict avoidance and good relationships were valued as much as the basic needs of survival. These extrapolated findings indicate that besides symptom management, food and housing security, psychosocial interventions for Rohingya refugees need to be grounded on ensuring a ‘good life’ for them based on respect, dignity, equality and autonomy. Interventions can similarly target social environments. White and Van der Boor (2021) had also proposed the CA to Formulating Experiences (CAFÉ) tool to understand how ecological factors at various levels can adversely or protectively affect the mental well-being of refugees. It is indeed a psychotherapeutic formulation considering the predisposing, triggering, perpetuating, situational and resilience factors within the marginalised populations. Consequent to that, interventions can be tailored in each of the sub-systems (Fig. 1), ex: support and psychotherapy (microsystem), family decision-making and community support groups (mesosystem), language-support, involvement of media, skill development and stigma-protection programmes (exosystem), and finally national and international rehabilitation-promoting and refugee-inclusive legislations (macrosystem). Many of these principles are included in the MHPSS, but further research and implementation in the Rohingya population remain as a test of time.

Whilst their larger fate hangs in the balance, community-level interventions such as sports and cultural activities can foster a sense of belongingness and provide a healthy emotional outlet. Suggested programmes include gatherings at mosques/community centres and participating in shared activities can help further consolidate their identity (Majeed, 2019).

At a family level, the interventions could look at minimising the transmission of trauma across generations. These include improving communication about grief and ensuring parents don't trivialise and compare the daily stressors their children endure to the trauma and violence of their own past. Group therapy has also been shown to be an effective tool to address trauma in asylees. Finally, at an individual level, apart from an individualised and need base mental health care service, other suggested interventions include normalising distrust, creating historical awareness and cultural competency (Majeed, 2019).

The role of grassroots organisations can be to provide these downstream services. They could include education programmes, health and safety awareness, and help with food/clothing collection and distribution.

Within India, the most important step could be to recognise the Rohingya as refugees and bring them under the ambit of the government. Although, this is majorly a politico-economic shift, its repercussions would lead to their emancipation in the social, economic and health domains. India has multiple psychiatric training centres, including tertiary care facilities in every zone, which train psychiatrists, psychologists and social workers. As a result, the number of MHP in India outnumber those in neighbouring Bangladesh or Myanmar. Hence, cross-nation collaboration is urgently called for. India can also draw upon its experience from successfully helping migrants transition in the past. From the times of immigrants who came into India during the Indo-Pak separation in 1947, to Tibetan and Bangladeshi refugees and the more recent Sri Lankan refugees, India has a history at helping them to draw upon (Saha, n.d.).

At the ground level, the provision of MHP who periodically visit and screen all the camp residents, followed by a referral to speciality centres, could be the first step. Tele-psychiatry also provides us with an opportunity to bridge the chasm between remote populations and specialists, and there is emerging evidence to support this claim (Hassan and Sharif, 2019; Soron *et al*., 2019). With the emergence of telemedicine guidelines in India during the COVID-19 pandemic, specialist service delivery can be optimised in this population (Dinakaran *et al*., 2021). Whilst provision of in-camp individual therapy and/or counselling services may place huge manpower demands, group therapy for victims of trauma and abuse may be helpful. Gender and age-based violence and sexual abuse are often under-reported and need to be addressed (Hutchinson, 2018). Sexual and reproductive rights preservation is crucial among adolescents, youth, women and gender minorities. Frequent crises and stressors affect their psychosexual health, reproductive safety, marriage practices, sexuality-related knowledge, attitudes, and practices (Ainul *et al*., 2018). Fostering sexual health and gender respect have been suggested to prevent gender-based discrimination and violence (Hutchinson, 2018). Especially in such a violence and chaos-prone population, inclusive psychosocial support programmes in case of emergencies have also been suggested, which involve crisis interventions, mobile mental health teams and psychological first aid (Ozen and Ziveri, 2019). This is based on the MHPSS principles, targets those with psychiatric disabilities, is multidisciplinary and focuses on reducing psychosocial morbidity.

Further enhancing the contributions of volunteers from within the community who refer cases to health works must not be overlooked (Dyer and Biswas, 2019). The role of traditional healers (discussed before) cannot be over-emphasised here. They play a vital role in Rohingya culture and enable help-seeking as well as stigma reduction. Understanding this socio-culturally sanctioned help-seeking behaviour and active involvement of the community traditional healers can enable culturally-tailored psychosocial interventions.

Education by health workers could be broadened to include information about mental health, thus countering stigma and improving attitudes towards mental illnesses. However, no intervention can address the social suffering of the Rohingyas until grounded in their voices. In line with the UNHCR Inter-Agency Standing Committee (IASC guidelines), inclusion and participation of individuals from their community are warranted in decision-making, both for health services and policies. Lived experiences can help tailored interventions (‘they know what they need’) (Inter-Agency Standing Committee (IASC), 2007).

Finally, this population also provides a reservoir of opportunity for research. Research can serve a dual purpose. While cross-sectional studies are available, which provides a ‘snapshot’ of their distress, longitudinal population-based studies and cross-country research will enable understanding of their psychosocial challenges at larger levels. Further, ethnographic and phenomenological studies provide a golden opportunity to explore their coping and navigation through adversities. They will have vital policy implications.

Planning and implementing research projects in these communities will not only serve to broaden our knowledge and understanding but also help provide the necessary funding and manpower to plug the existing gap in mental health infrastructure. The MHP community, however, need to be mindful of consent and informed decision making in this vulnerable community, safeguarding rights, justice and ethics during research.

## Conclusion

The Rohingya population is a prototype of the global refugee crisis. It is fundamental to consider them as a heterogeneous population based on the socio-political circumstances of various nations they reside in, leading to unique psychosocial issues and unmet needs. This paper provides an overarching view of the same. The ongoing widespread discrimination, prejudice, displacement, and violence contribute to their social suffering, which leads to systemic challenges and multi-levelled needs. Estimates of mental disorders, while being essential information, estimates of mental disorders fail to do justice to the more complex multi-faceted psychosocial challenges of the Rohingyas that cannot be merely viewed through a ‘medicalised’ lens. Attention and research need to focus on the trauma of displacement, loss of social identity, culturally specific idioms of distress, internal violence and marginalisation, all of which contribute to ‘minority stress’ in this community. The existing MHPSS interventions need to be improvised and implemented to understand how the Rohingyas navigate the challenges and develop resilience amidst adversities. Many of their mental health challenges appear as ‘survival threats’ which need integration into public health to improve their quality of life. The MHPs play a crucial role in this regard both as practitioners and advocators of mental health in this population, with intersectoral collaboration at various levels. The multi-tiered interventions for their mental well-being need to be contextually appropriate, culturally relevant and tailored into policies. The Rohingya crisis may be dire, but it provides yet another opportunity to re-integrate them into the community through principles of social justice, inclusion and health equality. It falls on our collective conscience to identify the vulnerabilities and chalk out a plan of action to possibly resolve this 200-year-old saga.
